# Increase of nitrosative stress in patients with eosinophilic pneumonia

**DOI:** 10.1186/1465-9921-12-81

**Published:** 2011-06-17

**Authors:** Kanako Furukawa, Hisatoshi Sugiura, Kazuto Matsunaga, Tomohiro Ichikawa, Akira Koarai, Tsunahiko Hirano, Satoru Yanagisawa, Yoshiaki Minakata, Keiichiro Akamatsu, Masae Kanda, Manabu Nishigai, Masakazu Ichinose

**Affiliations:** 1 Third Department of Internal Medicine, Wakayama Medical University School of Medicine, 811-1 Kimiidera, Wakayama, Wakayama 641-0012, Japan; 2 Chest M.I., Inc., 3-6-10 Hongo, Bunkyo-ku, Tokyo 113-0033, Japan

**Keywords:** Alveolar nitric oxide, corticosteroid, fractional exhaled nitric oxide, inducible type of nitric oxide synthase, 3-nitrotyrosine

## Abstract

**Background:**

Exhaled nitric oxide (NO) production is increased in asthma and reflects the degree of airway inflammation. The alveolar NO concentration (Calv) in interstitial pneumonia is reported to be increased. However, it remains unknown whether NO production is increased and nitrosative stress occurs in eosinophilic pneumonia (EP). We hypothesized that nitrosative stress markers including Calv, inducible type of NO synthase (iNOS), and 3-nitrotyrosine (3-NT), are upregulated in EP.

**Methods:**

Exhaled NO including fractional exhaled NO (FE_NO_) and Calv was measured in ten healthy subjects, 13 patients with idiopathic pulmonary fibrosis (IPF), and 13 patients with EP. iNOS expression and 3-NT formation were assessed by immunocytochemistory in BALf cells. The exhaled NO, lung function, and systemic inflammatory markers of the EP patients were investigated after corticosteroid treatment for 4 weeks.

**Results:**

The Calv levels in the EP group (14.4 ± 2.0 ppb) were significantly higher than those in the healthy subjects (5.1 ± 0.6 ppb, p < 0.01) and the IPF groups (6.3 ± 0.6 ppb, p < 0.01) as well as the FE_NO _and the corrected Calv levels (all p < 0.01). More iNOS and 3-NT positive cells were observed in the EP group compared to the healthy subject and IPF patient. The Calv levels had significant positive correlations with both iNOS (r = 0.858, p < 0.05) and 3-NT positive cells (r = 0.924, p < 0.01). Corticosteroid treatment significantly reduced both the FE_NO _(p < 0.05) and the Calv levels (p < 0.01). The magnitude of reduction in the Calv levels had a significant positive correlation with the peripheral blood eosinophil counts (r = 0.802, p < 0.05).

**Conclusions:**

These results suggested that excessive nitrosative stress occurred in EP and that Calv could be a marker of the disease activity.

## Introduction

Eosinophilic pneumonia (EP) is an inflammatory lung disease characterized by the infiltration of eosinophils into the alveolar region and interstitium of the lung [1,2]. The accumulation of eosinophils into the lung in EP is reported to be induced by the excessive production of eosinophil chemotactic mediators including interleukin-5 (IL-5) [3,4], IL-18 [5], and granulocyte-macrophage colony-stimulating factor (GM-CSF) [4]. Eosinophils contain a number of preformed mediators and cytotoxic enzymes within cytoplasmic granules [6]. The most abundant preformed substances are major basic protein (MBP), eosinophil cationic protein (ECP), eosinophil derived neurotoxin (EDN), and eosinophil peroxidase (EPO) [6]. In general, these mediators cause desquamation and destruction of the epithelium, and lead to airway and alveolar damage and lung dysfunction [6]. Eosinophils also release superoxide anion, leukotrienes, and various kinds of cytokines that cause tissue injury and inflammation. Thus, eosinophils are believed to play a major role in the pathogenesis of eosinophilic lung diseases. However, another mechanism of lung inflammation occurring in EP remains unknown.

Eosinophils are key cells to induce airway inflammation of asthma [6], whereas oxidative/nitrosative stress was recently reported to be related to the pathogenesis of asthma [7,8]. Infiltrated eosinophils in the airways of asthma express the inducible type of nitric oxide (NO) synthase (iNOS), which generates higher amounts of NO relative to the constitutive type of NOS (cNOS) [9]. Eosinophils also possess nicotinamide adenine dinucleotide (NADPH) oxidase complex. Activated NADPH oxidase catalyzes oxygen to superoxide anion, which enters further redox pathways to generate hydrogen peroxide in the presence of superoxide dismutase, or hydroxyl and nitrogen dioxide radicals, after combining with NO [10]. NO rapidly reacts with superoxide anion to form highly reactive nitrogen species (RNS) such as peroxynitrite [11]. Since excessive RNS cause tissue injury and stimulate the production of proinflammatory cytokines and chemokines [8,12], nitrosative stress could be one of the factors responsible for airway inflammation in asthma [8,13]. It has not been elucidated yet whether nitrosative stress may occur in the lungs of patients with EP.

In corticosteroid-naive asthmatic patients, the exhaled NO levels are markedly elevated compared to those in healthy subjects [14]. It has been reported that the levels of fractional exhaled NO (FE_NO_) have significant correlations with eosinophilic inflammation [15] and airway hyperresponsiveness in asthma [16]. Recently, the local NO production could be determined by partitioning exhaled NO into the alveolar NO concentration (Calv) and the conducting airway wall flux of NO (JawNO), and the Calv levels were found to reflect the NO production at the lung parenchyma [17]. In fact, the Calv levels were elevated in patients with alveolitis including hypersensitivity pneumonitis and idiopathic pulmonary fibrosis (IPF) compared to those in asthmatics and healthy subjects [18]. If the Calv levels in EP are elevated, it might indicate that the excessively generated NO in the lung parenchyma induces nitrosative stress in EP.

The aim of this study, therefore, was to investigate NO production and the resulting nitrosative stress in EP. Furthermore, we examined whether the Calv levels changed during treatment with systemic corticosteroid to assess whether it can be a marker of the response by treatment. To accomplish this, healthy subjects and patients with IPF and EP were enrolled in the current study. We investigated the exhaled NO production including FE_NO _and Calv. iNOS expression and 3-nitrotyrosine (3-NT) formation, a footprint of RNS production, were assessed in the cells of bronchoalveolar lavage fluid (BALf) as nitrosative stress markers. We investigated the correlation between the exhaled NO levels and lung function or systemic inflammatory markers such as peripheral blood eosinophil counts and C-reactive protein (CRP). In addition, we assessed whether the magnitude of reduction in Calv was correlated with that in systemic inflammatory markers during corticosteroid treatment.

## Methods

### Subjects

Thirteen patients with EP, 13 patients with IPF, and 10 healthy subjects took part in the present study after giving written informed consent. All subjects were never- or ex-smokers. None of the subjects had been treated with systemic and/or inhaled corticosteroids. All the patients with EP had acute or chronic respiratory symptoms including cough and sputum, pulmonary infiltrates on chest X-ray test and CT scan. They had pulmonary eosinophilia diagnosed by transbronchial lung biopsy (TBLB) according to the criteria of American Thoracic Society [2]. The patients with EP had no recurrent episodes of wheezing, no previous history of atopy and had never been diagnosed with bronchial asthma. IPF was diagnosed by pulmonary function tests, chest X-ray, and CT scan according to the criteria of the American Thoracic Society [19]. These patients had had restrictive ventilatory defect, interstitial infiltrates such as ground glass opacity and honey combing on CT scan and had no clinical history of exposure to hazardous environmental agents. Healthy subjects had normal lung function, no abnormality in chest X-ray, and no respiratory symptoms. None of the subjects had had a respiratory tract infection in the month preceding the study. This study was approved by the ethics committee of Wakayama Medical University.

### Study design

Exhaled NO including FE_NO _and Calv were measured according to previous studies [17,20]. All subjects received pulmonary function tests by CHESTAC (Chest Co. Ltd., Tokyo, Japan). All EP patients underwent bronchoscopy. One IPF patient and one healthy subject also received bronchoscopy. Eight of 13 EP patients were treated with systemic predonisolone (1mg/kg/day) for four weeks, with the dose of corticosteroid decreased gradually and finally discontinued within the first 6 months according to the previous guideline [2]. The treatment was started as a part of the routine treatment. Clinical symptoms, chest X-ray findings and the results of the blood examination were appropriately assessed to evaluate the effects of corticosteroid treatment. After corticosteroid treatment for 4 weeks, the exhaled NO and the pulmonary function were assessed. Peripheral eosinophil counts and CRP levels were also investigated.

### Measurement of FE_NO _and Calv

FE_NO _was measured according to the criteria of the American Thoracic Society using a chemiluminescence analyzer (NA-623N; Kimoto Electric, Osaka, Japan) [20]. Briefly, the subject exhaled at a positive constant mouth pressure (15 cmH_2_O) from the total lung capacity level. The FE_NO _was determined at a constant flow rate of 50 ml/s. The exhaled flow rates were verified at 50, 100, 175, and 370 ml/s to calculate the Calv according to a previous study [17]. For each flow rate, at least two technically adequate measurements were performed. Calv and JawNO were calculated with the two compartment model of NO exchange [17]. Moreover, we calculated the corrected Calv using the trumpet model with axial diffusion [21].

### BAL and TBLB

Fiberoptic bronchoscopy, BAL and TBLB were performed as previously described [22]. The obtained BALfs were immediately centrifuged at 650 x *g *for 5 min at 4 °C. The supernatant was stored at -80 °C. The cells in the BALfs were counted by hemocytometer and the cell viability was determined by the trypan blue exclusion method. A 100 μl aliquot of the suspension was placed into the cups of a Shandon 4 cytocentrifuge (Shandon Southern Instruments, Sewickley, PA) and five slides were obtained from each sample. The cell differential count was made after the staining with Diff-Quik (Sysmex Co.Ltd., Kobe, Japan). The obtained lung tissues were fixed by 10% formalin and sliced 4 micrometer thickness. The slides were stained by hematoxylin and eosin staining and photographed with a digital camera (DMX-1200C; Nikon, Tokyo, Japan) under ×400 magnification.

### Immunocytostaining

Immunocytostaining for iNOS or 3-NT in BALf cells was performed as previously described [23]. Briefly, the cells were fixed in 4% paraformaldehyde fixative solution for 30 min at room temperature. After blocking endogenous peroxidase, the samples were incubated with blocking reagents containing 0.3% Triton-X (Dako Cytomation, Kyoto, Japan) to reduce non-specific binding of antibodies for 30 min at room temperature. The cells were incubated with anti-iNOS rabbit antisera (1:200 dilution; Wako Pure Chemical Industries, Osaka, Japan) or anti-nitrotyrosine rabbit polyclonal antibody (1:100 dilution; Upstate Biotechnology, Lake Placid, NY) at 4 °C overnight. After being washed, the cells were incubated with secondary antibodies (ENVISION polymer reagent, Dako Cytomation, Kyoto, Japan). The diaminobenzidine reaction was performed and followed by counterstaining with hematoxylin. The cells were viewed by microscopy (E-800; Nikon, Tokyo, Japan) and photographed with a digital camera (DMX-1200C; Nikon, Tokyo, Japan) under ×400 magnification. Two investigators examined more than 500 cells and counted iNOS or 3-NT immunopositive cells without prior knowledge of the disease. The mean values were used for analysis.

### Collection of exhaled breath condensate (EBC)

The EBCs were collected from the healthy subjects and patients with IPF and EP using a condenser, which permitted the noninvasive collection of condensed exhaled air by freezing it to -20°C (Eco-screen; Jaeger, Hoechberg, Germany) according to the criteria of the European Respiratory Society [24]. The obtained EBC was stored at -80°C until later assay.

### Cytokine measurements in EBC

The expression of 42 different cytokines in EBC was investigated by Human Cytokine Antibody III kit (Ray Biotech Inc., Norcross, GA) according to the manufacturer's instructions.

### Statistical analysis

Data were expressed as mean ± SEMs. Experiments with multiple comparisons were evaluated by one way ANOVA followed by the Scheffe's test. Spearman's correlation analysis was performed to assess the correlation. Probability values of less than 0.05 were considered significant.

## Results

Ten healthy subjects, 13 patients with IPF, and 13 patients with EP took part in the present study. The characteristics of the study subjects are given in Table 1. Although the patients with IPF and EP had significantly lower vital capacity % predicted (%VC) than the healthy subjects, and the patients with IPF had significantly lower total lung capacity % predicted (%TLC), functional residual capacity % predicted (%FRC), residual volume (RV), RV % predicted (%RV), and diffusion lung carbon monoxide % predicted (%DL_CO_) than the patients with EP, there was no significant difference in other values of lung function among three groups. Although eosinophil counts in BALf were not so high in some patients with EP in this study, eosinophil infiltration into the alveolar septa was observed in the lung tissues from all EP patients (Additional file 1, Figure S1).

**Table 1:** Characteristics of the study subjects

	HS	IPF	EP
Number (M/F)	10(4/6)	13(12/1)	13(7/6)
Age (yrs )	60.9 ± 4.5	69.5 ± 1.9	63.2 ± 3.6
Smoking status (never-/ex-/current smoker)	(6/4/0)	(1/12/0)	(8/5/0)
VC (L)	3.20 ± 0.18	2.94 ± 0.23	2.57 ± 0.25
%VC (%)	108 ± 3.6	87.0 ± 5.8*	86.9 ± 7.0*
FEV_1.0 _(L)	2.54 ± 0.16	2.40 ± 0.16	2.08 ± 0.18
FEV_1.0% _(%)	80.5 ± 2.6	80.8 ± 1.6	82.4 ± 2.5
TLC (L)	N.D.	4.04 ± 0.32	4.61 ± 0.45
%TLC (%)	N.D	73.7 ± 6.0	96.5 ± 7.1^†^
FRC (L)	N.D.	2.40 ± 0.14	2.86 ± 0.24
%FRC (%)	N.D	74.9 ± 6.5	97.8 ± 6.9^†^
RV (L)	N.D.	1.34 ± 0.13	1.90 ± 0.19^†^
%RV (%)	N.D.	66.4 ± 9.9	115 ± 14^†^
%D_LCO _(%)	N.D.	66.7 ± 5.4	91.5 ± 11^†^
%D_LCO_/V_A _(%)	N.D.	73.6 ± 5.4	86.7 ± 6.1

HS = healthy subject; IPF = idiopathic pulmonary fibrosis; EP = eosinophilic pneumonia; VC = vital capacity; %VC = VC % predicted; FEV_1.0 _= forced expiratory volume in one second; TLC = total lung capacity; %TLC = TLC % predicted; FRC = functional residual capacity; %FRC = FRC % predicted; RV = residual volume; %RV = RV % predicted; %D_LCO _= diffusion lung carbon monoxide % predicted; %D_LCO_/V_A _= D_LCO_/alveolar volume % predicted; N.D. = not done. *p < 0.05 compared with the values of HS group; ^†^p < 0.05 compared with the values of IPF group.

### Exhaled NO levels in the study subjects

**Figure 1:**
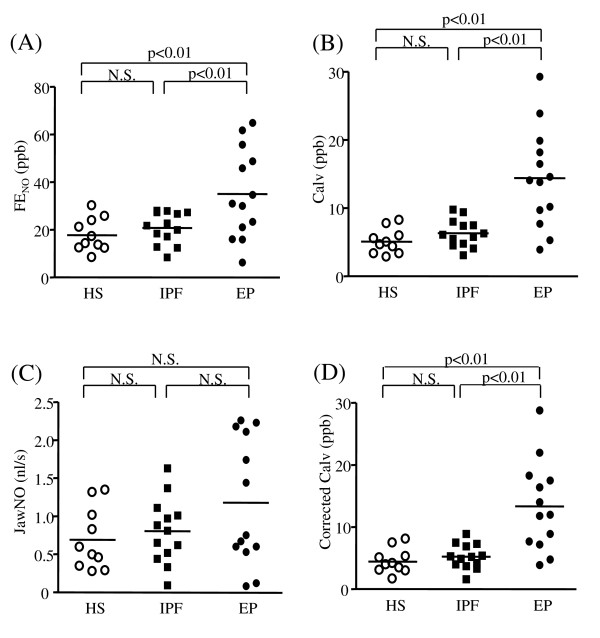
**Exhaled nitric oxide (NO) levels in the study subjects**. Panels show the fractional exhaled NO (FE_NO_) levels (A), the alveolar NO (Calv) levels (B), airway wall NO (JawNO) (C), and corrected Calv (D). Horizontal lines represent the mean value of the exhaled NO levels. HS = healthy subject; IPF = idiopathic pulmonary fibrosis; EP = eosinophilic pneumonia; N.S = not significant.

The FE_NO _levels in the EP group (35.0 ± 5.2 ppb) were significantly higher than in the healthy subject group (17.8 ± 2.2 ppb, p < 0.01) and the IPF group (20.8 ± 1.8 ppb, p < 0.01, Figure 1A). Because eosinophilic inflammation occurs in the lung parenchyma in EP, we speculated that the Calv levels in the EP group would be elevated compared to the other two groups. As we expected, the Calv levels in the EP group (14.4 ± 2.0 ppb) were markedly higher than in the healthy subject (5.1 ± 0.6 ppb, p < 0.01) and the IPF groups (6.3 ± 0.6 ppb, p < 0.01, Figure 1B). JawNO was also calculated with a two compartment model. There was no significant difference among the three groups (Figure 1C). To avoid the influence of contamination from NO produced in the airways to Calv, we also calculated the corrected Calv. The corrected Calv levels in the EP group (13.3 ± 2.0 ppb) were significantly higher than those in both the healthy subjects (4.5 ± 0.6 ppb, p < 0.01) and the IPF groups (5.3 ± 0.6 ppb, p < 0.01, Figure 1D).

### iNOS expression and nitrosative stress in EP

**Figure 2:**
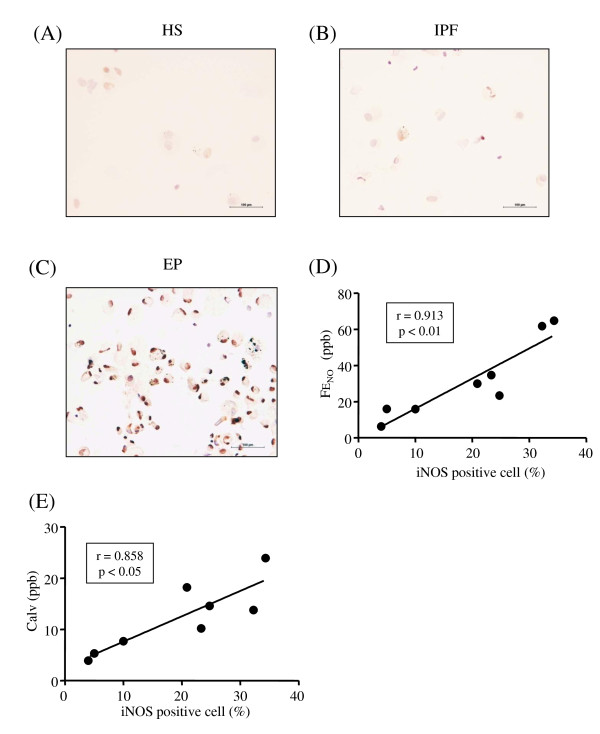
**Immunocytochemical detection of the inducible type of NO synthase (iNOS) in the bronchoalveolar lavage fluid (BALf) cells**. Representative photographs are shown in panel A (healthy subject: HS); B (idiopathic pulmonary fibrosis: IPF); and C (eosinophilic pneumonia: EP). iNOS immunopositivity in BALf cells is correlated with FE_NO _(D) and Calv levels (E). r is the correlation coefficient. The lines and p values correspond to the fitted regression equation.

**Figure 3:**
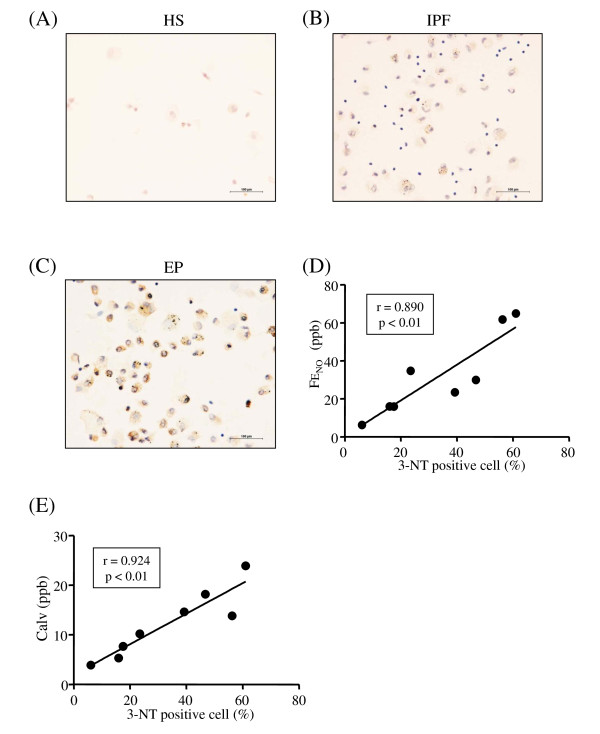
**Immunocytochemical detection of the 3-nitrotyrosine (3-NT) in the BALf cells**. Representative photographs are shown in panel A (healthy subject: HS); B (idiopathic pulmonary fibrosis: IPF); and C (eosinophilic pneumonia: EP). 3-NT immunopositivity in BALf cells is correlated with FE_NO _(D) and Calv levels (E). r is the correlation coefficient. The lines and p values correspond to the fitted regression equation.

**Figure 4:**
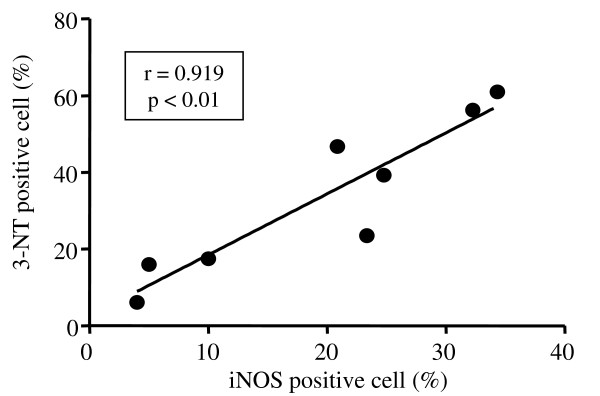
**Correlation between iNOS immunopositivity and 3-NT immunopositivity in the BALf cells**. r is the correlation coefficient. The lines and p values correspond to the fitted regression equation.

Cell differential counts in the BALf of the study subjects are listed in Additional file 2, Table S1. To investigate the source of increased NO production in the exhaled air from the patients with EP, we performed immunostaining for iNOS in the BALf cells. More iNOS positive cells were observed in the patients with EP than in the healthy subject and IPF patient (Figure 2A-C, Additional file 3, Table S2). There were significant positive correlations between the proportion of iNOS positive cells and the FE_NO _levels (r = 0.913, p < 0.01, Figure 2D), JawNO levels (r = 0.869, p < 0.05), or the Calv levels (r = 0.858, p < 0.05, Figure 2E). More 3-NT positive cells were also observed in the patients with EP than in the healthy subject and IPF patient (Figure 3A-C, Additional file 3, Table S2). There were significant positive correlations between the proportion of 3-NT positive cells and the FE_NO _levels (r = 0.890, p < 0.01, Figure 3D), JawNO levels (r = 0.790, p < 0.05), or the Calv levels (r = 0.924, p < 0.01, Figure 3E). The proportion of iNOS positive cells was significantly correlated with that of 3-NT positive cells (r = 0.919, p < 0.01, Figure 4).

### Correlation between the exhaled NO levels and lung function or inflammatory markers

We examined the correlation between the exhaled NO levels and the values of lung function and systemic inflammatory markers in the patients with EP before systemic steroid treatment (Table 2). There were significant correlations between the Calv levels and VC (r = -0.670, p < 0.05), %VC (r = -0.645, p < 0.05), forced expiratory volume in one second (FEV_1.0_) (r = -0.662, p < 0.05) or peripheral blood eosinophil counts (r = 0.658, p < 0.05).

**Table 2:** Correlation between the exhaled nitric oxide levels and lung function, systemic inflammatory markers and eosinophils in BALf

	FE_NO_		Calv	
	r	p value	r	p value
VC (L)	- 0.270	0.372	- 0.670	0.012*
%VC (%)	- 0.111	0.718	- 0.645	0.017*
FEV_1.0 _(L)	- 0.248	0.414	- 0.662	0.014*
FEV_1.0% _(%)	0.254	0.403	0.240	0.431
%D_LCO _(%)	- 0.057	0.853	- 0.316	0.272
%D_LCO_/V_A _(%)	- 0.258	0.395	-0.018	0.953
Eosinophils (/μl)	0.379	0.201	0.658	0.015*
CRP (mg/dl)	-0.358	0.229	-0.057	0.853
Eosinophils in BALf (%)	-0.183	0.638	-0.060	0.878

NO = nitric oxide; BALf = bronchoalveolar lavage fluid; FE_NO _= fractional exhaled NO; Calv = alveolar NO concentration; CRP = C-reactive protein. r = correlation coefficient, p values correspond to the fitted regression equation. *p < 0.05.

### Analysis of cytokine and chemokine profile in EBC

EBCs were obtained from nine healthy subjects, eleven IPF patients and nine EP patients. We examined the expression of 42 different cytokines in EBC using a cytokine assay method. The cytokine and chemokine profiling are summarized in Additional file 4, Table S3. There was no significant difference in their expression among the 3 groups.

### The effects of corticosteroid treatment on nitrosative stress in the patients with EP

To elucidate whether the exhaled NO levels in EP changes during systemic corticosteroid treatment, we measured the exhaled NO levels as well as lung function and systemic inflammatory markers before/after treatment with systemic corticosteroid. All patients' symptoms and chest radiographic findings were completely improved by corticosteroid treatment for 4 weeks. After corticosteroid treatment, the FE_NO _(44.1 ± 4.7 ppb vs 27.3 ± 2.1 ppb, p < 0.05) and the Calv levels (15.1 ± 2.4 ppb vs 6.90 ± 0.87 ppb, p < 0.01) were significantly reduced (Table 3). As expected, among the lung function tests, the VC (2.46 ± 0.38 L vs 2.96 ± 0.34 L, p < 0.01) and %VC (83.6 ± 11% vs 100 ± 11%, p < 0.01) values were significantly restored (Table 3). Peripheral blood eosinophil counts (584 ± 210/μl vs 45.4 ± 13/μl, p < 0.01) and CRP levels (1.91 ± 1.0 mg/dl vs 0.348 ± 0.29 mg/dl, p < 0.05) were also significantly reduced (Table 3). To determine whether the exhaled NO reflects the lung inflammation in EP, we investigated the correlation between the degree of reduction in the exhaled NO levels and those in the values of lung function and systemic inflammatory markers after corticosteroid treatment (Table 4). There was a significant positive correlation between the magnitude of the steroid-mediated reduction in the Calv levels and the peripheral blood eosinophil counts (r = 0.802, p < 0.05).

**Table 3:** Changes in the exhaled NO levels, lung function and systemic inflammatory markers during steroid treatment

	pre	post	p value
FE_NO _(ppb)	44.1 ± 4.7	27.3 ± 2.1	p = 0.021*
Calv (ppb)	15.1 ± 2.4	6.90 ± 0.87	p = 0.008**
VC (L)	2.46 ± 0.38	2. 96 ± 0.34	p = 0.008**
%VC (%)	83.6 ± 11	100 ± 11	p = 0.008**
FEV_1.0 _(L)	1.96 ± 0.24	2.12 ± 0.23	p = 0.11
FEV_1.0% _(%)	82.2 ± 3.9	78.2 ± 4.6	p = 0.47
%FEV_1.0 _(%)	82.0 ± 9.1	91.2 ± 9.0	p = 0.25
%D_LCO _(%)	92.4 ± 18	113 ± 15	p = 0.11
%D_LCO_/V_A _(%)	79.4 ± 6.8	85.3 ± 5.1	p = 0.47
eosinophil (/μl)	584 ± 210	45.4 ± 13	p = 0.008**
CRP (mg/dl)	1.91 ± 1.0	0.348 ± 0.29	p = 0.016*

pre = pre steroid treatment; post = post steroid treatment; p values compared with the values of pre steroid treatment. * p < 0.05, ** p < 0.01

**Table 4:** Correlation between the changes in the exhaled NO levels and those in lung function and systemic inflammatory markers after steroid treatment

	FE_NO_(post/pre)		Calv(post/pre)	
	r	p value	r	p value
%VC (post/pre)	-0.024	0.977	0.048	0.935
Eosinophils (post/pre)	0.108	0.793	0.802	0.022*
CRP (post/pre)	-0.524	0.197	-0.691	0.069

post/pre = post steroid value/pre steroid value; r = correlation coefficient; p values correspond to the fitted regression equation. * p < 0.05.

## Discussion

The present study demonstrated that the Calv levels in the patients with EP were significantly higher than those in the healthy subjects and the patients with IPF. We also demonstrated that more iNOS positive cells and 3-NT positive cells in the BALf were observed in EP than in IPF and healthy subject. The proportion of both the iNOS-positive cells and the 3-NT positive cells in the BALf was significantly correlated with the exhaled NO levels. Especially, the Calv levels had significant correlations with VC,%VC, FEV_1.0_, or peripheral blood eosinophil counts before steroid treatment. Systemic corticosteroid treatment reduced the Calv and the FE_NO _levels. The magnitude of the steroid-mediated reduction in the Calv levels was significantly correlated with that in the peripheral blood eosinophil counts. These results suggest that more nitrosative stress occurred in the EP patients compared to those in the IPF patients and Calv might be a marker of the response by treatment.

In inflammatory conditions, excessive NO was produced by iNOS as well as superoxide anion by NADPH oxidase or xanthine oxidase [8,11]. NO reacts with superoxide anion to produce the highly reactive RNS [11]. RNS are also generated via the H2O2/peroxidase-dependent nitrite oxidation pathway [25]. These RNS cause tissue damage due to active protease or toxic moieties released by stimulated inflammatory cells. RNS also augment plasma leakage and alter the function of several proteins by the nitration of tyrosine residues [8,26]. Furthermore, RNS augment tissue remodeling through the stimulation of nuclear factor-kappa B (NF-kB) - transforming growth factor-beta (TGF-β) pathway [27,28]. This is the first study to investigate oxidative and/or nitrosative stress in EP. In the current study, more 3-NT positive cells were observed in the BALf of EP patients, suggesting that more nitrosative stress occurred in EP. Because of the powerful inflammatory effects of RNS, nitrosative stress may be related to the inflammation that occurs in EP.

RNS, including NO and peroxynitrite derived from iNOS, have been reported to cause tissue inflammation in various kinds of diseases [8,29]. Although the precise mechanism is unknown, RNS may be involved in the pathogenesis of EP through the following mechanisms. First, endogenous NO could stimulate eosinophil migration in a rodent model because NOS inhibitors inhibit eosinophil infiltration into the tissues [13,30]. Moreover, Hebestreit et al. demonstrate that endogenous NO could prolong eosinophil survival induced by Fas ligand-induced apoptosis [31]. These findings suggested that RNS might play a key role in eosinophilic inflammation in EP. Second, RNS induce microvascular hyperpermeability [13] as well as tissue remodeling through matrix metalloproteinases (MMPs) activation and fibroblast-mediated tissue fibrosis [27,32]. Because EP is one of the interstitial lung diseases, the lung tissue remodeling observed in EP may be partially mediated by RNS. Nitrosative stress might be involved in the pathogenesis of EP, but further study is needed to clarify these mechanisms.

We demonstrated that the Calv levels in the EP patients were higher than those in the healthy subjects and the IPF patients, whereas there was no significant difference in the JawNO levels among the three groups. The JawNO levels in the EP group correlated with the iNOS positive cell counts. However, we also calculated the levels of the corrected Calv, which avoided contamination by the NO produced in the airways. The corrected Calv levels in the patients with EP were higher than in the other two groups, suggesting that the increase of exhaled NO (i.e. FE_NO_) in the EP patients could be attributed to increased NO production from the peripheral lung (i.e. Calv).

In the present study, there was a good correlation between the iNOS positive cells and the exhaled NO levels including Calv and FE_NO_. These findings suggest that iNOS might be the source of the exhaled NO in the patients with EP. According to the immunocytochemistory study, macrophages and granulocytes showed strong immunoreactivity suggesting that these cells may be the major source of NO production. Recently, Brindicci et al. demonstrated that both iNOS and neuronal NOS (nNOS) expression were enhanced in the lung peripheral tissues from chronic obstructive pulmonary disease (COPD) patients [33]. Therefore, the source of increased alveolar NO production (i.e. Calv) observed in this study could be mediated by iNOS and nNOS. Unfortunately, we could not obtain lung tissue from the patients, and we did not investigate nNOS and endothelial NOS (eNOS) expression in the BALf cells. It remains unclear which isoform of NOS is responsible for the elevated Calv levels. As shown in Figure 4, there was a very good correlation between the iNOS positive cells and the 3-NT positive cells suggesting that iNOS might be responsible for the RNS production. Since the mechanism for upregulation of iNOS is still unknown, further study is needed.

Corticosteroids have a number of anti-inflammatory actions including the suppression of iNOS expression [34]. In the current study, systemic corticosteroid treatment improved the clinical symptoms, chest radiographic findings, and inflammatory markers. It reduced the Calv levels almost to within the normal range. The reduction in the Calv levels might be due to the suppression of iNOS expression. Because nitrosative stress causes lung inflammation, the therapeutic effects of corticosteroid on EP may be mediated partially through the suppression of nitrosative stress. There were significant correlations between the Calv levels and lung function or peripheral blood eosinophil counts (Table 2). Interestingly, there was a good correlation between the magnitude of the steroid-mediated reduction in the Calv levels and that in the peripheral blood eosinophil counts (Table 4). These findings suggest that Calv may be a good biomarker of the disease activity in EP. Because Calv measurement is an easy and noninvasive method, it might be useful for assessing the degree of lung inflammation in EP.

Alveolar NO concentration (Calv [ppb]) is described by the following formula(1)

where V_NO,alv _[nl/s] is NO diffusing rate from tissue to alveolar air and DL_NO _[nl/s/ppb] is NO diffusing capacity from alveolar space to pulmonary vessels [18]. As DL_NO _is approximately 4* DL_CO _[18], the equation (1) can be rearranged to(2)

Hence, the values of Calv are affected by V_NO,alv _and DL_CO_. Calv can be increased because of the increased NO production in lung parenchyma causing increased NO diffusion to alveolar air (i.e. V_NO,alv_), or because of decreased diffusion of NO from the alveolar air to pulmonary blood stream caused by decreased alveolar NO diffusing capacity (i.e. DL_NO _= 4*DL_CO_). In the current study, the values of DL_CO _in the EP group were better than those in the IPF group (Table 1). Taken together, the "actual" NO production in the lung parenchyma appeared to be increased more in the patients with EP compared to the IPF patients.

Previous studies described that collecting EBC is a noninvasive and repeatable method, and useful for measuring airway inflammatory molecules in respiratory diseases including asthma [35] and COPD [36]. There was no difference in the expression of 42 cytokines and chemokines in EBCs (Additional file 4, Table S3), although the Calv levels were markedly elevated in the EP group compared to the IPF group and healthy subject group. Thus, measurement of Calv could be extremely useful for the assessment of lung inflammation in EP.

We used the IPF patients as disease controls in the current study because EP is classified as interstitial pneumonia. The current study is designed to address whether Calv could be a noninvasive method for the differential diagnosis of various interstitial pneumonias. As previously reported, nitrosative stress occurs in the airways of asthmatic patients [23]. In this study, the percentage of 3-NT immunopositive cells in BALf (33 ± 7%) from the EP patients was nearly the same as that in the induced sputum (29 ± 4%) from asthmatic patients [23]. Because the obtained samples differed between these two studies, it is not easy to compare the degree of nitrosative stress between EP and asthma.

As shown in Table 2, there were significant correlations between the values of Calv and those of VC,%VC, and FEV_1.0_. We expected that the Calv levels would have a correlation with%DL_CO _because eosinophilic inflammation is observed in the lung parenchyma in EP. Patients with EP sometimes have a restrictive ventilatory impairment. This is one possible explanation for the correlation between the Calv levels and%VC. In the current study, the actual values of FEV_1.0 _had a correlation with the Calv levels. This was an unexpected finding for us because the main site of inflammation in EP is the lung parenchyma, not the airways. There was no correlation between the Calv levels and the FEV_1.0 _values in previous studies [37,38]. Moreover, a correlation was observed between the Calv levels and the actual values of FEV_1.0_, not FEV_1.0%_. On the basis of these findings, the reason why the Calv levels had a correlation with the FEV_1.0 _values remains unknown.

We measured Calv only twice in this study. It would be interesting to examine if there was any correlation of Calv with the symptoms of the patients. However, it is difficult to assess the symptom scores in EP as well as asthma control test. There was a significant correlation between the changes in the Calv levels and the eosinophil counts after steroid treatment as shown in Table 4. We believe that Calv would be an extremely useful marker of the disease activity.

The limitations of the current study are as follows. First, we failed to collect BALf samples from patients with IPF and from healthy subjects. Because IPF is sometimes worsened by the procedure for obtaining BAL, we could not perform it. As for the healthy subjects, most refused the BAL examination. A previous study showed that low levels of iNOS as well as 3-NT formation were expressed in inflammatory cells of lung tissues from patients with the inactive stage of IPF and healthy subjects [39]. Our iNOS and 3-NT immunostaining data are compatible with those of a previous report [39]. Second, we could not obtain large size of lung tissues from the EP patients, and therefore could not investigate the expression of iNOS and 3-NT formation. Because airway and alveolar epithelial cells, endothelial cells, and vascular smooth muscle cells have been reported to express iNOS [8], these cells may also contribute to the nitrosative stress.

In summary, our data demonstrate that excessive NO production, presumably via iNOS, occurred in the patients with EP. The nitrosative stress markers were well correlated with the lung function and systemic inflammatory markers. Corticosteroid treatment improved the Calv levels as well as the clinical signs. The magnitude of the steroid-mediated reduction in the Calv levels was correlated with the peripheral blood eosinophil counts. Excessive nitrosative stress occurred in the patients with EP compared to the healthy subjects and the IPF patients and may induce the inflammation observed in EP because of the powerful proinflammatory effects of RNS. In addition, Calv could be a useful marker of the symptoms, severity and response to treatment in EP.

## List of abbreviations

BAL: bronchoalveolar lavage; Calv: alveolar NO concentration; EBC: exhaled breath condensate; EP: eosinophilic pneumonia; FE_NO_: fractional exhaled nitric oxide; iNOS: inducible type of nitric oxide synthase; IPF: idiopathic pulmonary fibrosis; NO: nitric oxide; 3-NT: 3-nitrotyrosine;

## Competing interests

The authors declare that they have no competing interests.

## Authors' contributions

KF carried out the data analysis and drafted the manuscript. HS and MI developed the study design and contributed substantially to the manuscript. KM, AK, TH, KA, and YM contributed to recruitment of the study subjects. HS and TI also carried out the data analysis. All other authors assisted with assessment of the data and interpretation. All authors contributed significantly to the development of the manuscript and all have seen and approved the final version and taken responsibility for the content.

## Supplementary Material

Additional file 1 **Lung tissues from the study subjects with eosinophilic pneumonia (EP) obtained by transbronchial lung biopsy**. Representative photographs show eosinophil infiltration into alveolar septa in the lung tissues from the patients with EP. The lung tissues from the three patients with EP are shown in panel A-C. Arrow heads indicate infiltrated eosinophils. Original magnification is ×400. Click here for file

Additional file 2 **Cell differential counts in the bronchoalveolar lavage fluid from the study subjects**. Included the PDF file. Click here for file

Additional file 3 **Percentages of immunopositive cells in the bronchoalveolar lavage fluid**. Included the PDF file. Click here for file

Additional file 4 **Cytokine and chemokine profile in exhaled breath condensate**. Included the PDF file. Click here for file
