# Supplementary figures and images for: Increase of nitrosative stress in patients with eosinophilic pneumonia
Source: Respir Res. 2011 Jun 17;12(1):81. doi: 10.1186/1465-9921-12-81 (PMC3141419; doi:10.1186/1465-9921-12-81)

(A)

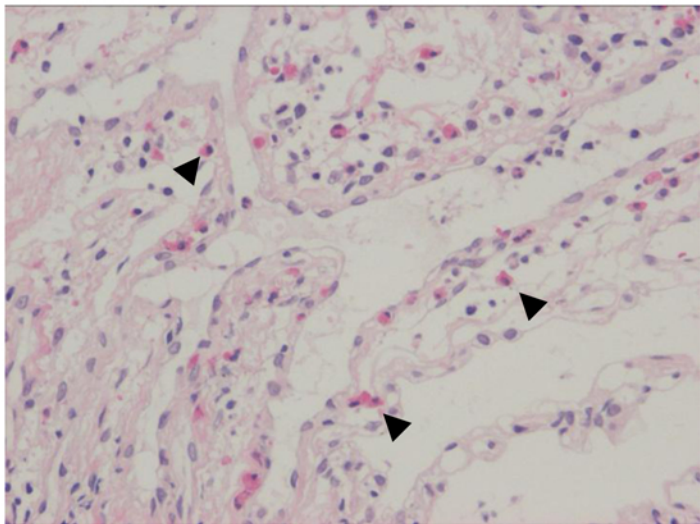

(B)

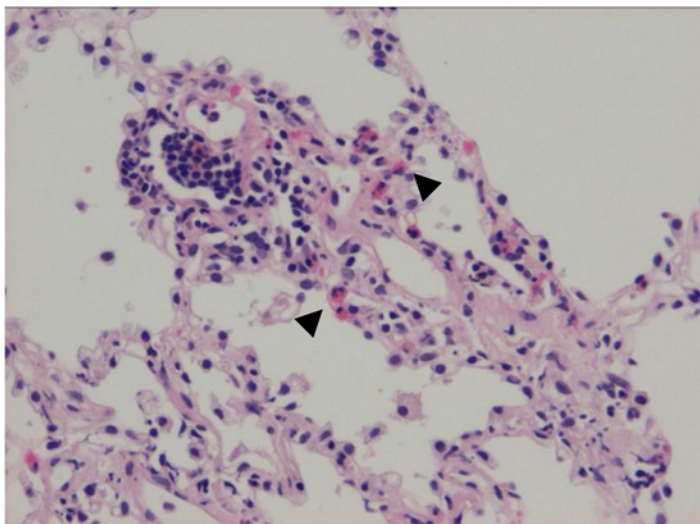

(C)

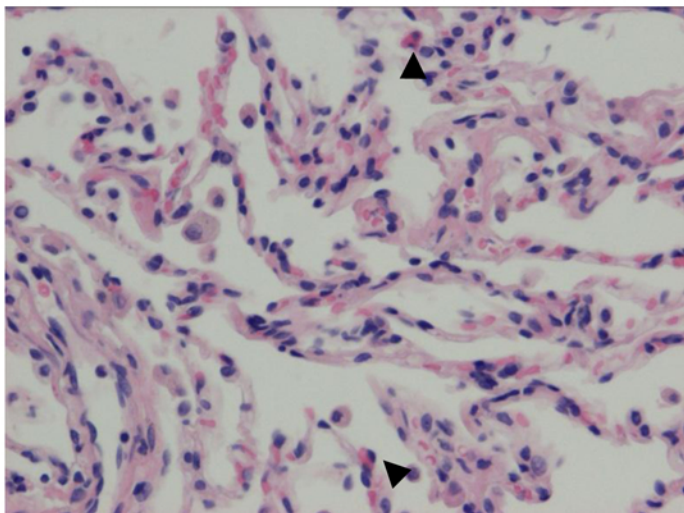

Supplement: Additional file 1 — Lung tissues from the study subjects with eosinophilic pneumonia (EP) obtained by transbronchial lung biopsy. Representative photographs show eosinophil infiltration into alveolar septa in the lung tissues from the patients with EP. The lung tissues from the three patients with EP are shown in panel A-C. Arrow heads indicate infiltrated eosinophils. Original magnification is ×400. [file 1465-9921-12-81-S1.PDF]
