# Supplementary material for: Increase of nitrosative stress in patients with eosinophilic pneumonia
Source: Respir Res. 2011 Jun 17;12(1):81. doi: 10.1186/1465-9921-12-81 (PMC3141419; doi:10.1186/1465-9921-12-81)
Supplement: Additional file 2 — Cell differential counts in the bronchoalveolar lavage fluid from the study subjects. Included the PDF file. [file 1465-9921-12-81-S2.PDF]

Additional file 2. Cell differential counts in the bronchoalveolar lavage fluid from the study subjects.

	number	WBC ($\times 10^5$/ml)	macrophages (%)	neutrophils (%)	lymphocytes (%)	eosinophils (%)
HS	1	2.8	73	1	26	0
IPF	1	2.2	55	8	36	1
EP	1	3.0	87	5	8	0
	2	1.0	68	3	26	3
	3	1.0	91	6	3	0
	4	4.5	36	3	54	7
	5	1.8	64	11	24	1
	6	2.8	77	1	22	0
	7	1.9	62	10	22	6
	8	6.8	26	21	34	9
	9	2.6	95	1	4	0

HS = healthy subject; IPF = idiopathic pulmonary fibrosis; EP = eosinophilic pneumonia; WBC = white blood cell count.
