# Supplementary material for: Increase of nitrosative stress in patients with eosinophilic pneumonia
Source: Respir Res. 2011 Jun 17;12(1):81. doi: 10.1186/1465-9921-12-81 (PMC3141419; doi:10.1186/1465-9921-12-81)
Supplement: Additional file 3 — Percentages of immunopositive cells in the bronchoalveolar lavage fluid. Included the PDF file. [file 1465-9921-12-81-S3.PDF]

Additional file 3. Percentage of immunopositive cells in the bronchoalveolar lavage fluid

		Total (%)	macrophages (%)	granulocytes (%)	lymphocytes (%)
HS					
	iNOS	8.5	9.1	0	0
	3-NT	26.0	27.3	0	0
IPF					
	iNOS	6.0	8.3	0	0
	3-NT	15.5	20.0	0	0
EP					
	iNOS	19.3±4.2	21.5±5.5	22.0±5.3	0
	3-NT	33.3±7.2	37.6±8.6	34.7±10	0

The data indicate mean ±S.E. All data were calculated as each immunopositive cell number / each total cell number. HS = healthy subject; IPF = idiopathic pulmonary fibrosis; EP = eosinophilic pneumonia; iNOS = inducible nitric oxide synthase; 3-NT = 3-nitrotyrosine.
