# Supplementary material for: Increase of nitrosative stress in patients with eosinophilic pneumonia
Source: Respir Res. 2011 Jun 17;12(1):81. doi: 10.1186/1465-9921-12-81 (PMC3141419; doi:10.1186/1465-9921-12-81)
Supplement: Additional file 4 — Cytokine and chemokine profile in exhaled breath condensate. Included the PDF file. [file 1465-9921-12-81-S4.PDF]

Additional file 4. Cytokine and chemokine profile in exhaled breath condensate

	HS(%)	IPF(%)	EP(%)		HS(%)	IPF(%)	EP(%)
angiogenin	4.4±0.3	4.1±0.2	4.3±0.2	IL-8	8.1±0.8	8.8±0.5	9.1±0.9
EGF	13.4±2.7	12.0±3.2	15.6±3.6	IFN	4.5±0.3	5.0±0.4	4.9±0.3
ENA78	4.5±0.4	4.8±0.3	4.7±0.4	Leptin	7.4±0.6	7.5±0.8	7.7±0.8
G-CSF	3.2±0.3	3.1±0.2	3.2±0.3	MCP-1	5.1±0.4	4.8±0.5	5.2±0.6
GM-CSF	3.2±0.3	3.1±0.2	3.2±0.3	MCP-2	4.2±0.2	4.1±0.3	4.4±0.3
GRO	5.9±0.4	6.8±0.4	5.7±0.4	MCP-3	4.3±0.4	4.0±0.2	4.1±0.2
GRO-α	4.5±0.4	4.6±0.2	4.4±0.3	MCSF	6.6±0.5	6.7±0.5	6.9±0.3
I-309	5.6±0.5	6.3±0.4	5.3±0.5	MDC	6.1±0.4	5.6±0.5	5.8±0.6
IGF-I	4.2±0.2	4.1±0.3	4.8±0.3	MIG	5.4±0.4	5.4±0.2	5.8±0.5
IL-10	7.9±0.5	8.5±0.6	6.9±0.4	MIP-1δ	5.9±0.9	6.1±0.5	6.0±0.8
IL-12p40p70	4.9±0.3	5.0±0.4	5.1±0.5	oncostatin M	7.6±0.5	8.1±0.7	9.2±0.9
IL-13	3.7±0.2	3.5±0.2	3.4±0.2	PDGF-BB	6.2±0.3	6.8±0.3	6.9±0.5
IL-15	4.2±0.2	4.2±0.2	4.2±0.2	RANTES	16.1±1.2	18.6±0.8	17.5±1.3
IL-1α	5.5±0.4	6.9±0.6	6.4±0.5	SCF	5.6±0.5	5.5±0.2	5.5±0.3
IL-1β	4.0±0.4	5.1±0.7	4.5±0.5	SDF-1	5.9±0.4	6.4±0.4	5.7±0.4
IL-2	4.6±0.4	4.0±0.3	3.9±0.6	TARC	6.7±0.6	6.6±0.5	6.1±0.4
IL-3	4.6±0.2	4.3±0.4	4.1±0.5	TGF-β_1	4.3±0.3	4.9±0.4	4.7±0.3
IL-4	3.9±0.2	3.9±0.2	3.9±0.3	thrombopoietin	4.5±0.4	4.2±0.3	4.9±0.4
IL-5	4.2±0.2	4.2±0.2	4.2±0.2	TNF-α	4.8±0.3	4.3±0.3	4.8±0.2
IL-6	4.2±0.4	4.4±0.2	4.0±0.2	TNF-β	5.9±0.4	5.8±0.8	6.7±0.8
IL-7	4.5±0.4	4.1±0.3	4.0±0.3	VEGF	5.4±0.4	5.2±0.3	5.4±0.3

All values indicate relative intensity compared to the positive control in EBC obtained from healthy subjects (HS, n = 9), idiopathic pulmonary fibrosis (IPF, n = 11) and eosinophilic pneumonia (EP, n = 9). All values are calculated by average of each cytokine or chemokine spot intensity level / average of the positive control spot intensity.

EGF = epidermal growth factor; ENA78 = epithelial-derived neutrophil attractant 78; G-CSF = granulocyte-colony stimulating factor; GM-CSF = granulocyte-macrophage colony stimulating factor; IGF-I = insulin-like growth factor; IL = interleukin; IFN = interferon gamma; MCP = monocyte chemotactic protein; MDC = macrophage-derived chemokine; MIG = monokine induced by interferon gamma; MIP-1δ = macrophage inflammatory protein 1δ; PDGF-BB = platelet-derived growth factor-BB; RANTES = regulated upon activation, normal T-cell expressed and secreted; SCF = stem cell factor; SDF-1 = stromal cell- derived factor 1; TARC = thymus and activation-regulated chemokine; TGF-β_1 = transforming growth factor-β_1; TNF = tumor necrosis factor; VEGF = vascular endothelial growth factor.
